# Environmental Transformations Enhancing Dignity in an Acute Psychiatric Ward: Outcome of a User-Driven Service Design Project

**DOI:** 10.1177/19375867221136558

**Published:** 2022-12-25

**Authors:** Ann Faerden, Christine Rosenqvist, Mats Håkansson, Eline Strøm-Gundersen, Åshild Stav, Jan Svartsund, Trude Røssæg, Nils Davik, Elfrida Kvarstein, Geir Pedersen, Ingrid Dieset, Anders Q. Nyrud, Harald Weedon-Fekjær, Kaja Misvær Kistorp

**Affiliations:** 1 Department of Acute Psychiatry, Oslo University Hospital, Norway; 2 User Representative Advisory Board, Department of Acute Psychiatry, Oslo University Hospital, Norway; 3 Lovisenberg Diaconal Hospital, Oslo, Norway; 4 EGGS Design Firm, Oslo, Norway; 5 BRANDL Architect Firm, Oslo, Norway; 6 Section for Personality Psychiatry and Specialized Treatment, Oslo University Hospital, Norway; 7 Norwegian University of Life Sciences; 8 Oslo Center for Biostatistics and Epidemiology, Research Support Services, Oslo University Hospital, Norway; 9 Oslo School of Architecture and Design, Norway

**Keywords:** dignity, hospital architecture, environment, seclusion, acute psychiatry, user involvement, innovation, service design

## Abstract

**Objectives:**

The goal of the current project was to enhance the feeling of dignity for patients in the seclusion unit in an acute psychiatric ward through environmental design changes and to evaluate the effect of the refurbishment.

**Background:**

Treating people with dignity is essential in all health-related work and important for our mental health. Hospital architecture and design signal values that can promote dignity. Patients who must spend time in seclusion are at their most vulnerable mental state and the often worn-down like environment can challenge the feeling of dignity. How environmental design can promote dignity in seclusion units have not been studied.

**Methods:**

To reach suggestions for design changes enhancing dignity, we used service design that included a broad user group. The effect of design changes was evaluated by a questionnaire answered by the nursing staff during a 4-week period pre- and post refurbishment and included a control group.

**Results:**

The design concepts agreed upon were a welcoming atmosphere, contact with nature, room for privacy, close contact with staff, and a designated smoking area inside the unit. The evaluation found that the environmental design changes significantly supported the patients in their situation and the staff in their work.

**Conclusion:**

We conclude that dignity design concepts are highly applicable also in an acute psychiatric setting and improve the situation of secluded mental health patients, which is much needed. Findings align with other environmental changes in psychiatric wards that improve the patients’ well-being and reduce aggression.

## Background

Hospital architecture and design signal values that can promote health (Connellan et al., 2013; Ulrich et al., 2008). Treating people with dignity is an essential value in all health-related work (Askitopoulou & Vgontzas, 2018), and dignity is important for our mental health (Caughey, 2018; Saxena & Hanna, 2015). Dignity is a universal concept, encompassing worth, respect, and autonomy (Webster, 1976). It is also an individual feeling experienced in interaction with our social settings and environment (Jacobson, 2007).

Patients admitted to an acute psychiatric ward are severely ill, some need treatment without their own consent, and some are in need of care in specialized seclusion rooms or units (Fisher, 1994; Steinert et al., 2010). These seclusion units are often more worn down than the rest of the ward and more reminiscent of prisons than healthcare facilities (Csipke et al., 2016; Liddicoat, 2019). This, combined with the loss of freedom, represent a challenge to human dignity (Chambers et al., 2014; Gustafsson et al., 2014; Prinsen & van Delden, 2009).

Seclusion is an old method used in mental health. The aim is to reduce stimulus and prevent harm because the patients are severely psychotic, manic, or agitated and with threats of violence toward oneself and others. Seclusion most often takes place in a special designated room or unit in a separate area within a locked ward. Countries regulate the practice of seclusion differently, but for most it is regulated within a Mental Health Act (Niveau, 2004; Steinert et al., 2010). Time spent in seclusion may vary from a few days to weeks to sometimes months. The reduction of stimulus from the environment is often reflected in naked walls, small windows, white paint, sparse lighting, and no room for activities or visitors. Prevention of harm is managed through sparsely furnished rooms. Units used for seclusion often bear the history from former patients with scratches and scribbles on the walls giving the impression of decay over time (Niveau, 2004). On these grounds, seclusion units are criticized by international bodies, the patients, family members, and staff (Askew et al., 2019; Niveau, 2004). Despite this continuing criticism, society and healthcare authorities have not been able to replace seclusion with other means (Bowers et al., 2017; Niveau, 2004; Oliver, 2021; Ukom, 2020). Therefore, it is up to each healthcare facility to provide seclusion under the best conditions possible.

Dignity in healthcare has mostly primarily focused on the delivery of care (Lindwall & Lohne, 2020) and much less on environmental aspects that may enhance and support the feeling of dignity (Baillie et al., 2009). How hospital architecture and environment interact with staff and patients have been studied in both general and psychiatric hospitals (Brambilla et al., 2019; Chrysikou, 2019; Connellan et al., 2013; Ulrich et al., 2008). Studies aiming to improve seclusion have focused on clinical interventions (Dahm et al., 2017; Väkiparta et al., 2019) and the role of the physical environment (Oostermeijer et al., 2021; Väkiparta et al., 2019; van der Schaaf et al., 2013). We have found one study of recent date addressing the effect of specific design elements in a secluded unit (van der Venne et al., 2021), but no study that explores how environmental design features may enhance dignity in seclusion units.

### The Current Study

The study started in 2012 upon the initiative from the staff at Ward 2 (W2), Department of Acute Psychiatry Oslo University Hospital (DAP-OUS), Norway, after having received criticism over the years from patients and family members. They characterized the environment in the seclusion unit as “prison-like” and undignified and asked how anybody could get well in such an environment. Correspondingly, the staff claimed that these physical surroundings worked against their efforts to provide good care. The environment signalized low investment in mental illness, an outdated psychiatric practice and a lack of user involvement. The project initially intended to paint the walls, but through collaboration with the Oslo School of Architecture and Design (AHO) it evolved into a user-driven service design research project. The main goal was to improve the feeling of dignity by changing the physical environment in such a way that it supported the patients in their situation and the staff in their work. We here report the design concepts aimed to enhance dignity and the evaluation of the refurbishment according to these concepts.

***The main goal was to improve the feeling of dignity by changing the physical environment in such a way that it supported the patients in their situation and the staff in their work.***

## Method

### Description of the Ward 2 and the Seclusion Unit

The DAP-OUS serves an adult population of 220.000 in Oslo municipality, with a total of 800–900 admissions per year. Approximately 60% are admitted to compulsory mental healthcare. The DAP-OUS has a total of 47 beds. All patients are admitted through a reception ward (W1) of which 50% are discharged after 1–3 days and those in need of longer stay are transferred from W1 to one of the four other wards, including W2. All five wards are locked and have a separate seclusion unit within the ward with one to four patient beds. Patients in the seclusion unit have no access to common rooms, the level of security is strengthened, and the patient/staff ratio is higher.

The W2 treats approximately 120 patients per year with mainly psychotic illness. The W2 has eight patient rooms, of which three were in the seclusion unit. Approximately a third of the patients at W2 spend time in the seclusion unit which can last a day, up to a week or several weeks. There are three nursing shifts per 24 hr (day shift: five staff members, evening: three, and night: two). At least, two of the daytime staff are allocated to the seclusion unit. Treatment is organized in multiprofessional teams around each patient. For patient to be allocated to the seclusion unit, the Norwegian Mental Health Act requires a legal decision on why such measures are required. Patients in the seclusion unit always have access to staff. They cannot be isolated except for a short period of maximum 2 hours in the case they represent a severe danger to others and only until staff gain control of the situation. Before the transformation, only one room in the seclusion unit had a separate bathroom, and adequate consultation, visiting, or activity rooms were lacking and rendered little opportunity for patients to regulate contact with others, provide shielding, or meet privacy needs. The patient rooms had naked walls, sparse lighting, and furnishing (Figure 2, Photo 1). Meals were mainly served in the room or in the corridor. The unit had a room for indoor smoking (Photo 4).

### Participants and the Service Design Process

A broad range of users—patients, family members, and staff—are relevant in a project designing hospital environments aiming to promote dignity (Borg et al., 2009; Jacobson, 2007). Methods for user involvement range from giving advice, being member of the project and research groups, through focus group discussions, and the use of service design (Borg et al., 2009). This project was conducted using service design as the main approach. The project defined users in a broad term including those traditionally defined as users such as patients, family members, and user representatives. In addition, we defined health staff members working with patients (nurses, social workers, psychologists, and psychiatrists) and healthcare managers as users since they are “users” of the ward environment. This was important to create ownership and commitment to the process. To get a broader understanding of different users’ needs and environmental design elements which may enhance dignity, we invited personnel from two other secluded units: the Department of Acute Psychiatry at Lovisenberg Diaconal Hospital (DAP-LDH) and Attendo Paulus Nursing Home (APNH). They were asked because they had recently refurbished and changed the interior of their seclusion unit and thus could contribute with experience from their’ refurbishment.

**Figure 1.**
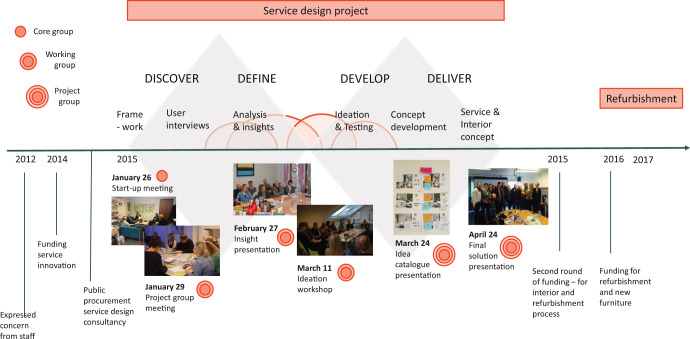
Time frame for the project from start to end.

The project consisted of three groups, the core group, working group, and project group (Figure 1). The core group consisted of six persons: the project manager from the DAP-OUS, two service designers, the user representative from DAP-OUS, and a health worker from DAP-OUS and DAP-LDH. The core group was responsible for organizing the workshops and securing progress according to the plan (Figure 1). A total of 20 people participated in the working group. It included the core group; an additional user representative from DAP-LDH; different healthcare workers from DAP-OUS, DAP-LDH, and APNH; and two interior architects (Figure 1). The working group took part in the design process from beginning to end, and provided insight, ideas and concept feedback. In addition to the working group, the project group included two health managers from DAP-OUS. Altogether, 22 people took part in the project (Figure 1).

The user representatives were members of the User Representative Advisory Board at DAP-OUS and DAP-LDH. They were part of the working group from the very start of—and throughout the project. The user representatives in the advisory board represent different Mental Health Organizations in Norway, and their main role is to secure the representation of patients’ needs and desires. In addition to the user representatives, people with patient experience and their family members were involved in the Discovery phase (Figure 1) through qualitative interviews. A total of six patients and family members were interviewed. This was conducted by the service designer in the beginning of the project to discover and explore user needs.

Service design is a systematic, academic discipline combining design, anthropology, process engineering, management, and psychology (Julier & Moore, 2009; Manzini, 2011; Stickdorn & Schneider, 2011). In the process of creating new designs, the focus is on the needs of people involved (human centricity), considers the complexity of all affected actors (cocreation), and involves them in a developmental and iterative process (Sleeswijk Visser, 2013). The service design method thus aims to capture experiences happening over time across various touch points within the service (Clatworthy, 2011, pp. 15–28; Holmlid, 2009; Meittinen & Koivisto, 2009; Segelstrøm, 2013). The current project draws on the double-diamond design process (Figure 1), a model developed by the British Design Council in 2015 (Counsel, 2015).

Figure 1 demonstrates the different design phases in this project. In the *discover phase,* aims were to gain insight into the context, current situation, and needs of all affected actors. The *define phase* covered identification of what might be designed, idea generation, and development of initial concepts. The *develop phase* provided further idea generation and testing of the chosen concept through prototypes. The *deliver phase* covered final adjustments and testing of the concept design solutions.

The project had its first meeting in January 2014 (Figure 1). Environmental design solutions were presented after 12 weeks. It took another year to get the financial support. The planning of the refurbishment started in June 2016, remodeling started in February of 2017 and finished 17 weeks later in June 2017 (Figure 1).

### Evaluation of Design Changes Enhancing Dignity

The evaluations took place during a 4-week period prior to the implementation process (March 2015) and 3 months after the refurbishing was finished (November 2017). We did not find a previously developed questionnaire focusing on environmental dignity (Heuzenroeder et al., 2020), and we developed two questions (Q) to assess the effect of the environmental design changes. The intent was to ask both patients and staff, but no patients were able to consent to research during the evaluation period due to their mental condition. Therefore, only staff took part in the formal part of the evaluation. The Qs asked were the following: Q1: “Do you feel that the physical environment in the seclusion ward supports the needs of your patients in their situation today?” Q2: “Do you feel that the physical environment of the seclusion ward has been supportive of your work as staff today.” The questions also included information on threats and violence during the shift. The Qs had five possible answers rated on a 1–5 scale (1 = *not at all*, 2 = *very little*, 3 = *somewhat*, 4 = *to a large degree,* and 5 = *all the time*). The staff at the seclusion unit at DAP-LDH took part as a control group of the evaluation since no refurbishment took place at this ward during the evaluation period. The questionnaire was filled out at the end of each shift.

### Ethics

The service design project and evaluation were approved by the data protection official at OUS (2015/2326 and 2015/2326). Participation required informed consent. Participation was partly anonymous with no names or birth numbers. Staff Qs included information on gender, age, education, and years of experience. These were considered relevant information which could impact responses.

### Statistical Methods

Descriptive data on staff background are given in frequency, percentages, median, and mean with standard deviation and range. For the evaluation of environmental change, we approximated the ordinal questionnaire scores using a linear regression model. To accommodate the repeated measurements from staff overlapping between the evaluations, we applied a linear regression mixed model. Uncertainty was described using 95% confidence intervals. Effect sizes (ESs) describing the difference between mean Q1 and Q2 scores before and after refurbishment were computed using Cohen’s *d* (Cohen, 1988) where *d* ≤ .2 is small, *d* ≤ .5 medium and d ≤ .8 a large ES. For robustness analysis, we adjusted for both gender and violence. Analyses were performed using SPSS Statistics Version 27.

## Results

### Environmental Design Concepts Enhancing Dignity and Design Solutions

The service design project concluded that the design concepts and environmental effects supporting dignity would be found in a welcoming atmosphere of calmness and modern design, view and contact with nature, creation of good spaces with room for privacy, various activities and meeting others, shielding of noise from other patients’ suffering, close contact with staff, and a smoker’s room in the unit. Keeping the indoor smoking room was up for debate, but the user representative was firm and their statement of “being severely ill was not the time to quit smoking” weighed in.

The above conclusions were generated as loose ideas in the discovery phase and transformed in the define phase into concrete design concepts and environmental effects. Design solutions to the different dignity concepts were proposed by the interior architect in the *define phase* and decided upon in the *deliver phase* (Figure 1). All ideas of what would enhance dignity from the discovery phase are incorporated into the final solution. The project group agreed that the main solution incorporating the concepts of dignity was the design of a wooden oak frame with multiple functions (Photos 2 and 3). The frame was intended to serve the need for both privacy and autonomy, different activities and contact with nature, both by the outside view and by the texture of solid wood. Other design concepts aiming to create a welcoming atmosphere and other forms of contact with nature were the choice of light wall colors characteristic of Nordic architecture and big images of nature (Nordic landscapes) placed on the walls in the corridor, one of the common rooms and smoking-room (Photos and 6–8). To further emphasize nature’s importance, the flooring was changed to a wooden-like outlook, with built-in heating for comfort.

To support the feeling of privacy and control over personal belongings, all rooms had a cupboard and three of the four patient rooms had bathrooms. Bathroom tiles had an earthly color matching the floors and other coloring. The unit was expanded in outline giving space for two common rooms. A soundproof door in the wall between them rendered the possibility for flexible use ensuring privacy and confidentiality when needed, and with the door open, more room for different activities (Photo 5). The common rooms served as therapeutic meeting rooms, for family visits, and for dining and activities. To show respect for patients’ needs a new smoking room was built with better ventilation and a chair to relax in. The staff working station was placed inside the unit to improve the contact between staff and patients. The furniture was of modern design to signal change and modern attitudes.

The project lasted 5 years from the initiation of the idea to the completion of refurbishment (Figure 1). All the main design solutions were incorporated in the refurbishing. Walls were torn down and the functions of rooms were reorganized. After the refurbishment, the seclusion unit included four patient rooms, three with private bathrooms, two common rooms, and a working room for staff. No patient rooms had other patient rooms next to it to shield from noise.

### Evaluation of the Refurbishing

#### Participants of the evaluation

A total of 618 questionnaires were returned from 159 different staff members at the two different seclusion units at DAP-OUS and DAP-LDH during a 4-week period in March 2015 and November 2017. All days had at least one returned questionnaire from both sites, with 10 at the most. There were more female employees among the OUS staff, more years of experience and a higher level of education compared to DAP- LDH. Age and employment status were similar at the two sites (Table 1).

**Table 1.** Description of the Staff at DAP-OUS and DAP-LDH.

	2015		2017	
Sociodemographic variables	DAP-OUS ^a^	DAP-LDH ^b^	DAP-OUS	DAP-LDH
Female staff (%)	86	35	65	17
Age: median and range	33 (24–66)	35 (23–56)	32 (22–58)	31 (23–51)
Work shifts (%)				
Day shift	53	56	42	49
Afternoon shift	39	27	34	38
Night shift	8	15	23	12
Employment (%)				
Permanent	80	75	73	70
Time based	20	25	27	31
Education				
High school	3	6	3	8
Nursing school	12	25	29	41
Nurse + specialist	67	46	37	15
Other	18	23	31	36
Years experience				
<1 year	2	22	3	8
1–4 years	15	8	35	38
4–8 years	12	24	15	17
8–10 years	24	0	5	4
>10 years	47	46	42	33

^a^ Staff from Oslo University Hospital. ^b^ Staff from Lovisenberg Diaconal Hospital.

### Results of Evaluation of Change at W2

Figure 3 presents % answers to the Q1: “Do you feel that the physical environment takes care of the patient’s needs in their situation today?” By merging the answers that were scored 4 and 5, 9.9% at DAP-OUS answered to a large degree/all the time in 2015 as compared to 75.0% from LDH. In the postchange evaluation, 74.9% of the staff at OUS and 67.2% at DAP-LDH answered to a large degree/all the time. The mean score of the physical environment support for the OUS patients increased from 2.6 to 3.9 from 2014 to 2017 (Table 2a). Linear mixed model analyses showed a large and significant increase in score of 1.38 at DAP-OUS and estimated by Cohen’s *d* to be of a large ES (Table 2b). There was no significant change in mean score at DAP-LDH.

**Figure 2.**
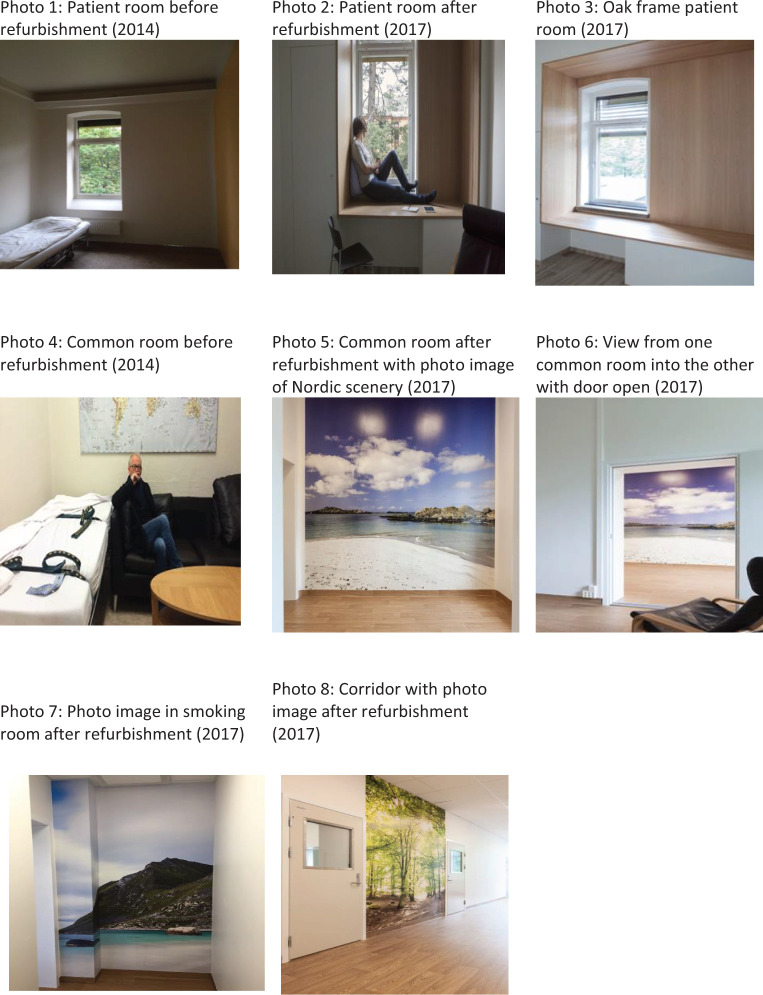
Photos 1–8: From the seclusion unit W2, OUS before and after refurbishment.

**Figure 3.**
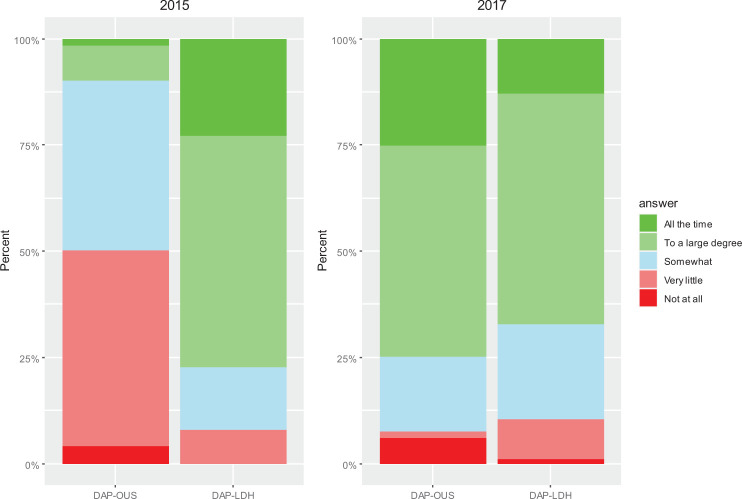
Results of the evaluation from 2015 and 2017. Q1 (staff): Do you feel that the physical environment in the secluded unit supports the needs of your patients in their situation to-day?

**Table 2.** Mean Reported Scores and Estimated Changes of Physical Environment Support; Before and After Transformation of the Environmental Design at DAP-OUS Between 2015 and 2017.

(a) Mean Reported Scores					
	Q1: Reported Physical Environment Support the Patient			Q2: Reported Physical Environment Support the Staff	
	DAP-OUS	DAP-LDH		DAP-OUS	DAP-LDH
	Mean (SD*)	Mean (SD*)		Mean (SD*)	Mean (SD*)
2015	2.57 (0.77)	3.82 (0.79)		2.78 (0.86)	3.88 (0.98)
2017	3.86 (1.01)	3.68 (0.86)		3.88 (0.98)	3.81 (0.79)
* SD = Standard deviation (b) Estimated Changes at the DAP-OUS After Refurbishment ^1^					
			Estimated change With 95% Confidence Interval	*p* Value (of Change)	Cohen’s *d*
Reported physical environment support:					
Q1: the needs your patient to-day			1.38 [0.90, 1.86]	<0.000	2.0
Q2: your work as staff to-day			1.19 [0.73, 1.64]	<0.000	1.7

^1^ The estimates are based on a mixed effects regression model to account for individual levels of reporting (among the repeated measurement data).

**Figure 4.**
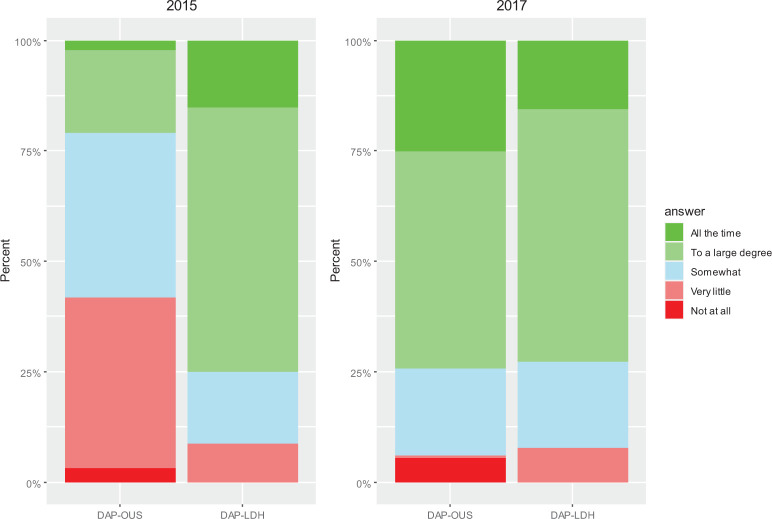
Results from the evaluation from 2015 and 2017: Q2 (staff): Do you feel that the physical environment of the secluded unit has been supportive of your work as staff today?

Figure 4 presents % answers to the Q2: “Do you feel that the physical environment in the seclusion ward supports the needs of the staff today?” In 2015, 20.9% of the staff at DAP-OUS and 80.4% at DAP-LDH answered to a large degree/the whole time to Q2. In the postchange evaluation in 2017, 74.2% at DAP-OUS and 72.8% at DAP-LDH answered to a large degree/the whole time. Mean score of the physical environment support for the staff at DAP-OUS increased from 2.8 to 3.9 during the study period (Table 2a). Linear mixed model analysis showed a large and significant increase in score of 1.2 at DAP-OUS, also of large ES (Table 2b). There was no significant change in mean score at DAP-LDS.

In models controlling for threats or differences in staff gender distribution, the reported results above were not significantly changed.

## Discussion

To the best of our knowledge, this is the first study focusing on environmental design elements that may enhance dignity in seclusion units in acute psychiatry and also in mental health in general (Aljunaidy & Adi, 2021). The project has also shown that through the use of service design, it is possible to include a large user group in designing the transformation of a health environment and perform a pre- and postevaluation of changes, both important elements to secure the validity of results that are often missing in design studies (Durosaiye et al., 2020; Oostermeijer et al., 2021). The concepts of a welcoming atmosphere, privacy, and contact with nature are in line with design elements that have been found to reduce seclusion and aggression (Borckardt et al., 2011; Ulrich et al., 2018; van der Schaaf et al., 2013). Together with our results, this supports the importance of such elements in the design of seclusion rooms and units in mental health hospitals.

### Concepts Representing Dignity

Human dignity is a challenging concept to evaluate as it is more than just a mere definition, and it varies according to the situation (Jacobson, 2007). To be able to study dignity in healthcare service, Nora Jacobson describes two forms of dignity: human and social dignity (Jacobson, 2007). Human dignity is the value that belongs to us all as humans while social dignity is “generated in the interaction between—and among individuals, collectives, and societies” (Jacobson, 2009). Further she divides social dignity into two types: dignity-of-self and dignity-in-relation (Jacobson, 2009). According to her results violation of dignity in healthcare service mostly appear in dignity-in-relation which takes place in the interaction with the delivery of service, through communication, behavior, and the physical environment (Jacobson, 2009).

Dignity in the health service has mostly focused on delivering of care (Lindwall & Lohne, 2020) and much less on how the environment interacts (Baillie et al., 2009). Governmental papers, reviews from academic institutions and architect firms address the importance of the physical environment and how it influences dignity, safety, and well-being, but none address specifically which elements support dignity (Baillie, 2009; Brambilla et al., 2019; Hammerstrøm, 2007; MASS Design Group, 2020; TSO, 2013; Ulrich et al., 2008). We have found eight studies addressing dignity in mental health services (A. Buchanan, 2015; Chambers et al., 2014; Gustafsson et al., 2013, 2014; Kogstad, 2009; Lindwall et al., 2012; Skorpen et al., 2016; Skorpen et al., 2014), where one address the importance of the environment (Chambers et al., 2014) and the other different aspects of communication and caretaking between staff and the patients. The concepts representing dignity in this study were a welcoming atmosphere, contact with nature, and room for privacy reflecting the importance of interaction with the surroundings and corresponds to the dignity-in-relation as defined by Jacobson. On the other hand, the dignity of self can be understood as need for privacy showing respect for the individual and feeling of intrusion. The design element best encompassing all this was found to be an oak frame around the window (Photos 2 and 3). The same “framing in” or alcove element can be seen in some newly built hospitals such as the acute psychiatric ward at St. Olavs Hospital in Trondheim, Norway, the psychiatric hospital in Slagelse, Denmark, and in the planning of a new general hospital in Stavanger, Norway (Stavanger, 2020). We have not found a description of why these hospitals have chosen such a design element, but both St. Olavs—and Slagelse Hospital focused on patient-centered care and the healing architecture (DOGA, 2019; Simonsen & Duff, 2020).

The use of the elements of nature in hospitals and other health environments has been given some attention with respect to general health benefits (Bratman et al., 2019), on reduction of stress (Collado et al., 2017; Mcsweeney , 2015; Totaforti, 2018), and positive psychological effects (Nyrud & Bringslimark, 2010; Nyrud et al., 2013). The current COVID-19 situation may add to the understanding of the relation between nature and dignity for users of a seclusion unit as many have been “in seclusion” from others. One important relief from the pandemic has been access to nature (Kaplan Mintz et al., 2021; Ugolini et al., 2020). The present study contributes to existing research on the possible effects of elements of nature (Hartig et al., 2014), this time from a psychiatric ward, a context different from the former studies focusing on somatic health services.

Most studies on how physical environment may influence patients are from general or forensic wards (Borckardt et al., 2011; Curtis et al., 2013; Karlin & Zeiss, 2006; Lundin & Caira, 2018; McCurdy et al., 2015; Papoulias et al., 2014; Sheehan et al., 2013; van der Schaaf et al., 2013). Many of these studies find that the same design elements as our study of feeling of privacy, separate bathroom, and a view of nature have a positive influence. But we have found only two studies focusing on seclusion units (Vaaler et al., 2005; van der Venne et al., 2021). Vaaler and colleagues (2005) found that an ordinary home design had a positive effect on many patient’s well-being, especially women, but with no change in violent episodes. Van de Venne (2021) and colleagues found small positive changes in the experience of seclusion in an enriched environment compared to seclusion in a regular seclusion room. These two studies focused on different design elements than in our project and point to the need for more studies on what elements can improve the situation for patients in secluded units.

This study is unique in the sense that it was carried out in a seclusion unit in an acute psychiatric ward where safety measures have been in the forefront of forming the physical environment (Chrysikou, 2019; Curtis et al., 2013). Healthcare facilities are important as they reflect the city and region’s health and welfare system and represent the platform where social, environmental economic, and medical interact (Brambilla et al., 2019). Therefore, it is of importance that a dignified environment is integrated into this context (R. Buchanan, 2001; Jacobson et al., 2009). Using service design with its human-centered focus and users with experiences from the mental healthcare service, our study indicates that design concepts are important for a dignified physical environment in a seclusion unit in acute psychiatry. However, more studies are needed to explore whether these concepts are of universal use, which could be of importance for improving the much-needed enhancement of dignity for mental health institutions worldwide (Shepley & Song, 2014).

### Evaluation of Environmental Design Changes

The goal of this project was to suggest environmental dignity concepts, implement them, and evaluate the effect. Studies on environmental design changes tend to be criticized because they seldom have control groups, and few have a pre- and postevaluation of the effect from staff or the patients (Connellan et al., 2013; Durosaiye et al., 2020). The study shows that the staff considered the new physical environment largely supportive to both the patients in their situation and supportive for their work. This is in line with what the Health Building Notes from the Department of Health in the UK for Adult Acute Mental Health Units (TSO, 2013) state about how a good physical environment is “a crucial element in the delivery of positive therapeutic outcomes for service users, their safety and the safety of staff.” These results are further supported by the results from the evaluation at DAP-LDH that served as a control group where there was no significant change in mean value. The study therefore adds to the growing literature that emphasize the importance of the physical environment in reinforcing treatment goals and positive expectations (Chrysikou, 2019; Connellan et al., 2013; Karlin & Zeiss, 2006). The influence on how the environment can support the feeling of dignity is mostly theoretical. Our study supports for such an influence in that the evaluation included a large number of staff, staff from every shift, changes were found to have a large positive effect, and evaluation took place over a 4-week period and with a control group at another hospital where no refurbishing took place.

### User-Driven Project

In addition to the tangible and visual results, the experiences from this project also gave important new input to understanding of “user participation.” The demand for user participation in designing the new ward made it necessary to rethink the idea of users’ role in service development. While the idea of “users’ participation” in service development is not new (Csipke et al., 2016; Lindberg et al., 2019; Millar et al., 2016; Romm et al., 2019), this study adds to the growing experience of the importance of user’s role in service development, both among users, users’ representatives, and service providers (Liddicoat et al., 2020). An example of this is the acceptance of the smoker’s need for dignity that signifies the service design’s central ideology: “the users’ needs are the most important contributors to the progress.” Despite the policy of a smoke-free hospital, the project managers saw the need for a dignified retreat area for those patients still smoking when being in a seclusion unit. This illustrates the importance and challenges of user-driven projects.

## Strengths and Limitations

The strength of the project is that it includes a broad user group from three different institutions, changes are evaluated, and a solid approach was used to find solutions. The limitation is that the evaluation in the form of a questionnaire only included the nursing staff since we underestimated that patients were not able to consent during the evaluation period. Also, the evaluation was by only two questions and did not directly evaluate dignity, but the changes agreed upon by the users to enhance dignity. Also, we did not assess health effects such as reduction in violence, days spent in the seclusion unit for the patients, and days away from work for the staff due to work conditions which are important health outcomes when evaluating hospital architecture and design.

## Conclusions

This project is unique in its inclusion of users, its focus on architectural design concepts that can enhance dignity in a psychiatric hospital, its use of service design, and with pre- and postevaluation of the changes. The design changes were found to have a large effect in supporting the patients and the staff. The design elements enhancing dignity of a welcoming atmosphere and view of nature are of common sense and much the same found to increase well-being and reduce aggression and which ultimately will reduce the need to keep patients in seclusion. The design concepts have the potential to be universally applied and find local and culturally adopted solutions. The cost of change was low and should inspire hospital management to refurbish worn down psychiatric wards which are criticized for its undignified interior. The project took 5 years to complete and was dependent on grants. The time frame signals that changes from bottom and up takes time and can be challenging to implement.

## Implication for Practice

- Environmental design concepts can enhance the feeling of dignity in seclusion units for mental health patients.
- Environmental design can support the staff in their work in an acute psychiatric setting.
- The design elements that represent dignity for mental health may be applicable to other health institutions.
